# Development of Vegetable Creams Enriched with Different Microalgae Species: A Study on the Physicochemical and Sensory Stability over Time

**DOI:** 10.3390/foods14071230

**Published:** 2025-03-31

**Authors:** Fabio Fanari, Josep Comaposada, Teresa Aymerich, Anna Claret, Luis Guerrero, Massimo Castellari

**Affiliations:** 1 Food Safety and Functionality Program, Institute of Agrifood Research and Technology (IRTA), 17121 Monells, Spain; teresa.aymerich@irta.cat (T.A.); massimo.castellari@irta.cat (M.C.); 2 Food Quality and Technology, Program Institute of Agrifood Research and Technology (IRTA), 17121 Monells, Spain; josep.comaposada@irta.cat (J.C.); anna.claret@irta.cat (A.C.); lluis.guerrero@irta.cat (L.G.)

**Keywords:** vegetable cream, Spirulina, *Chlorella vulgaris*, color, stability, sensory analysis, umami

## Abstract

Vegetable creams are a popular food with sensory characteristics (intense color, smooth texture, rich flavor) suitable for the inclusion of microalgae ingredients. Limited examples of vegetable creams reformulation with microalgae are reported in the literature, and no research has focused on their stability. This study evaluates the quality parameters of heat-treated, high-protein vegetable creams formulated with Spirulina, *Tetraselmis chui*, and four different *Chlorella vulgaris* strains over an 8-month period. The investigation examines changes in physicochemical properties (color, moisture, consistency, pH, °Brix, syneresis), microbiological parameters, and sensory profile. Physicochemical results showed enhanced homogenization effects of microalgae, suggesting valuable technological applications. The sensory analysis highlights a general enhancement of umami and salty perception, with differences depending on the species considered. Yellow chlorellas were the least impactful in terms of flavor but require further investigation regarding their pronounced color influence. *Tetraselmis chui* altered the most the sensory profile with a strong fishy and shellfish flavor. Over time, color variation deserves attention since slight browning phenomena, with possible negative effects on consumer perception, were observed. Regarding sensory aspects, limited and no detrimental effects were detected over time in texture, taste, and smell. No adverse impact on shelf life was observed, suggesting applications in long-term storage foods.

## 1. Introduction

Vegetable creams are vegetarian meals typically elaborated by blending one or more vegetable ingredients. These products present a distinctive creamy-liquid texture and are often used as starters, side courses, or as ingredients in more complex dishes. The basic ingredients used to prepare these creams are tubers, bulbs, fruits, and herbal extracts. Furthermore, they can contain dried fruits, legumes, and other plant-based ingredients. Additional ingredients such as stabilizers, emulsifiers, sweeteners, and flavorings are sometimes also added to improve the techno-functional and sensory properties.

In recent years, vegetable creams have become increasingly popular especially due to their nutritional characteristics. Vegetables are rich in vitamins and minerals. A diet rich in fruits and vegetables can reduce the risk of cardiovascular diseases, prevent some types of cancer, lower the risk of eye and digestive problems, and have a positive effect on blood sugar, which can help keep appetite in check [1]. Other reasons that contributed to the increase in vegetable creams consumption are the growing demand for ready-to-eat products (according to current social habits), the boom in diets based on veganism, and the possibilities that these foods offer to innovate and develop new products (ingredients, tastes, process, packaging, etc.). The estimated value of fruit and vegetables produced in the EU reached €73.4 bn in 2022 [2], while the global soup market size was around €16.0 bn in 2019 and is expected to reach around €23.0 bn by 2032 [3]. This highlights a rising interest in these products. Consumers have also increased their demand for foods with high-protein content, made with few organic ingredients, more sustainable, and manufactured through less invasive production processes [4]. Microalgae-based ingredients can potentially cover this demand.

Microalgae are microscopic aquatic organisms that have gained considerable attention in the food industry. They are particularly interesting due to their high nutritional value, rich bioactive compounds, and potential eco-friendly cultivation [5,6,7]. *Arthrospira platensis* (commercially known as Spirulina) and *Chlorella vulgaris* (*C. vulgaris*), usually grown in freshwater, are the most well-known and used microalgae species in food. In recent times, they have also found applications in the feed sector [8,9,10]. Nevertheless, seawater species are calling attention due to their interesting content of omega-3 fatty acids, minerals, key trace elements, and the higher sustainable potential of their cultivation process [11].

Recently, researchers investigated the potential of incorporating microalgae into various food formulations, including bread [12], pasta [13] and bakery products [14], energy bars [15] and snacks [16], and meat analogs [17,18]. Vegetable creams are an appealing matrix for incorporating microalgae due to their intense, color, smooth texture, rich flavor, and versatility. These characteristics can help mask off-colors, odors, and tastes usually associated with microalgae inclusion [19].

Previous studies have highlighted the potential of microalgae to enhance the nutritional profile of vegetable creams. Muela et al. [20] added 3.3% *Chlorella vulgaris* and 0.2% *Ascophyllum nodosum* to a vegetable cream, achieving a “source of protein” status. They also assessed bioactivity and sensory acceptability. Boukid et al. [21] used spirulina, *C. vulgaris*, *Tetraselmis chui* (*T. chui*), and *Nannochloropsis oceanica* in creams at 1.5% and 3.0% levels to formulate high-protein vegetable creams, observing varying effects on nutritional properties, color, consistency, water content, and water-holding capacity. Lafarga et al. [22] enriched broccoli soups with Spirulina sp., *Chlorella* sp., or *Tetraselmis* sp. at 0.5% to 2.0% concentrations, noting significant changes in nutritional properties, color, pH, viscosity, and sensory analysis. However, none of these studies evaluated long-term storage stability, a critical factor in food products. Vegetable creams, as emulsion-based systems, are susceptible to changes in their physicochemical properties during storage. Factors such as lipid oxidation, phase separation, microbial growth, and enzymatic reactions can affect the stability of these products, leading to undesirable changes in texture, sensory profile, and appearance [23,24]. Microalgae inclusion can affect the oxidative stability of the food [25] and this should be taken into account to complement the study aiming at their development. Moreover, another important aspect, such as the sensory profile assessment, has also been underexplored. Microalgae sensory features are an important parameter to consider in food applications. In the article by Muela et al. [20], a group of 30 people evaluated the differences between a control cream and the specific formulation proposed with 2.2% of yellow colored *Chlorella vulgaris*. No differences were detected in the attributes considered: appearance, aroma, color, taste, texture, and overall acceptability. This suggests a potential application of *Chlorella* but does not give any input on other species nor a characterization of typical sensory features of this alga. In contrast, Lafarga et al. [22], using a similar evaluation, reported that microalgae inclusion significantly decreased acceptability, texture, flavor, visual appearance, and purchase intention even at just 0.5%. This decrease was proportional to the microalgae concentration. Therefore, a more detailed sensory assessment is needed to identify off-odors, colors, and flavors, helping the food industry improve sensory profiles and consumer acceptability.

Following the above discussion, the main objective of this work was to shed light on the effects of the inclusion of microalgae SCI on the quality of vegetable creams. Single-cell commercial ingredients (SCI) from microalgae species approved for human consumption according to the European Novel Food Regulation (EU) 2015/2283 [26] were used to formulate high-protein vegetable creams. The considered microalgae were: (i) Spirulina and (ii) *C. vulgaris* (four different strains) cultivated in freshwater, and (iii) *T. chui* cultivated in saltwater. The long-term storage quality of the reformulated creams was investigated by assessing changes in physicochemical properties (color, moisture content, consistency, pH, °Brix, syneresis), microbiological parameters, and sensory profile over 8 months.

## 2. Materials and Methods

### 2.1. Vegetable Cream Preparation

**Figure A1:**
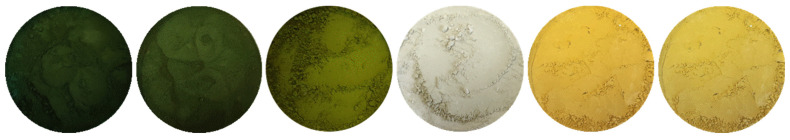
Microalgal single-cell ingredients used in the formulations. From left to right: Spirulina, *Tetraselmis chui*, *C. vulgaris* “Smooth”, *C. vulgaris* “White”, *C. vulgaris* “Honey”, and *C. vulgaris* GL3 strain.

Frozen vegetables (spinach, zucchini, chickpea, leek, broccoli, and chard) were bought from Geland (Girona, Spain). The chosen vegetables are commonly used in commercial vegetable cream preparation. Microalgae SCI were provided by Allmicroalgae Natural Products S.A. (Pataias, Portugal). The six microalgae SCI are commercial products consisted of spray-dried powders from microalgae biomasses produced in closed bioreactors, specifically: (i) *C. vulgaris* “Honey” (HC, yellow color), (ii) *C. vulgaris* “Smooth” (SC, pale green color), (iii) *C. vulgaris* “White” (WC, pale yellow color), (iv) *C. vulgaris* GL3 strain (GL3, yellow color), (v) Spirulina (SP, dark-green color), (vi) *T. chui* (TS, intense green color). In Figure A1, a picture of each microalgae ingredient is reported, to help understand the color differences. All these SCI products were commercially available, except GL3, an axenic strain of *C. vulgaris* isolated by random mutagenesis technique, which is part of the Allmicroalgae Natural Products S.A. culture collection. TS and SP were produced in heterotrophic conditions under natural light, while the other microalgae species were grown heterotrophically in the dark. Microalgae SCI were stored at 4 °C in opaque and vacuum packaging and used to elaborate the creams within 3 months. The rest of the ingredients (mineral water, sunflower oil, and salt) were purchased from a local supermarket. However, all ingredients used in this study are approved for consumption and therefore comply with current legislation on food safety and quality criteria.

Vegetable creams were prepared according to five different formulations, including the control recipe (control, without microalgae ingredients) and the five formulations with microalgae (see Table 1).

The amount of ingredient to be added for each microalgae species was calculated, according to the composition of each single-cell ingredient (see nutritional information reported in Table A1, Appendix A), to reach the claim “high in proteins” according to the CE regulation n° 1924/2006 [27]. The claim “high in proteins” can be declared on the label of a food product when at least 20% of its energy value comes from proteins.

**Figure A2:**
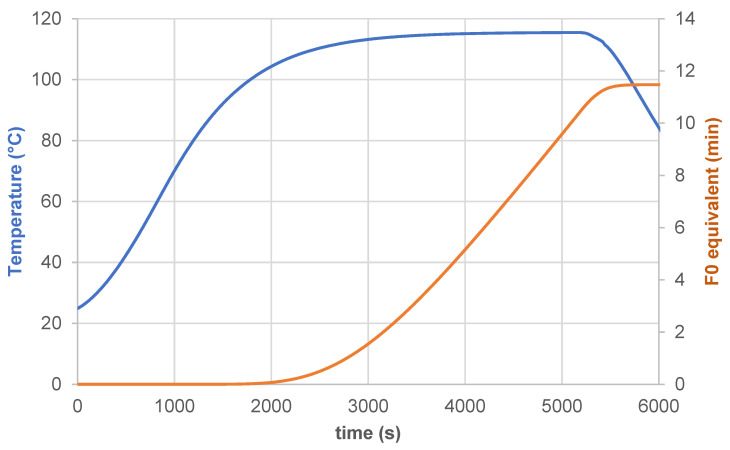
Average values of temperature evolution and equivalent lethality (F0) as a function of time during the sterilization process. Temperature data are represented in blue color, while F0 is reported in red color.

For the preparation of the creams, all ingredients were weighed, mixed, and cooked at 90 °C for 25 min with continuous mixing at 300 rpm in a cooking robot (Thermomix^®^, Vorwerk, Wuppertal, Germany). After that, the product was homogenized for 75 s by progressively increasing the mixing speed from 2000 to 7600 rpm. The creams were packaged in 200 mL glass bottles with hermetic caps, left to cool down to 20 °C in a cold-water bath, and finally heat-treated in an autoclave (Ilpra Systems, Mataró, Spain). The sterilization program used for the autoclave is detailed in Table A2 (Appendix A). To evaluate the effectiveness and lethality of the process, the F0 value, equivalent time (in min) at the reference temperature of 121.1 °C, of the process was calculated using Equation (1):

(1) $$F0=\sum \Delta t\times 10^{\frac{T-121.1{}^{\circ}C}{z}}$$

where *T* is the actual temperature that was measured inside the cream during the process (in °C), Δ*t* is the temperature sampling time interval that was 20 s, and the *z*-value is the temperature change needed to achieve a tenfold reduction in the D-value, which is the time required at a specific temperature to reduce the microbial population by 90%. The *z*-value was set at 10 °C as common practice for many processes. According to the temperature measured during the process, a value of total equivalent time at 121.1 °C (F0) of 11.48 min was achieved. The graphic of the temperature-lethality profile for one product taken as an example could be observed in Figure A2 (Appendix A). After sterilization, bottles were stored at room temperature in a warehouse.

### 2.2. Vegetable Creams Characterization

The samples (n = 21) of vegetable creams (three different batches for each formulation) were characterized at time 0, immediately after their production, and after 8 months of storage in darkness at room temperature (20 °C). This helps understand how the product’s quality and characteristics might change under typical storage conditions.

#### 2.2.1. Physicochemical Parameters

Moisture content (MC%) was assessed by drying the samples in a forced-air oven (J.P. Selecta, Abrera, Spain) at 105 °C to a constant weight for 24 h. pH was measured at 25 °C using a Crison pH25 (Crison Instruments, Barcelona, Spain). Syneresis (Sy%) was performed by centrifuging the samples in a Beckman Avanti JXN-30 (Beckman Coulter Inc., Brea, CA, USA) centrifuge equipped with a JA-25.50 rotor. A total of 30 g of cream was processed at 14,500 rpm (approx. 25,000× *g*) for 30 min at 4 °C. The Sy% was calculated weighting the supernatant and calculating the percentage over the initial sample (g supernatant/100 g cream) [28]. Soluble solid content (°Brix) was estimated with a handheld refractometer (Atago, ATC 1E, Tokyo, Japan) at 20 °C. For all the previously described analyses, three replicates of each sample were carried out.

The samples’ color was evaluated with a CR-600d colorimeter (Minolta Co., Osaka, Japan) by measuring CIELab parameters [29]. Measurements were performed using a CM-A124 Zero Calibration Box (Konica Minolta Inc., Osaka, Japan), an opaque black container suitable for color measurements of liquids, which was filled with approximately 50 mL of the sample. Total color difference (ΔE) from the control sample was calculated using the CIE76 formula (Equation (2), based on the Euclidean distances between colors in CIELab space):

(2) $$\Delta E=\sqrt{{\left(L_{0}-L\right)}^{2}+{\left(a_{0}-a\right)}^{2}+{\left(b_{0}-b\right)}^{2}}$$

where L*, a*, and b* are the CIELab parameters of the cream sample under study, and L_0_, a_0_, and b_0_ are the L*, a*, and b* measured for the control sample. To define a unique reference set of parameters, L_0_, a_0_, and b_0_ were determined as average values of the three replicates. Data were extracted using Color Data Software SpectraMagic™ NX CM-S100w software Version 2.8 (Konica Minolta Inc., Osaka, Japan). Each sample was scanned three times.

Measures of consistency were carried out using a Bostwick consistometer (ZXCON-CON3, PCE Instruments Ibérica S.L., Albacete, Spain). The sample chamber of the device was filled with 100 mL of product, and then the gate of the chamber was released to allow vegetable cream to flow. The Bostick measurement value was estimated by recording the distance (cm) traveled by the sample after 30 s [30,31]. A longer distance indicates a thinner consistency, while a shorter distance indicates a thicker consistency. A phone camera was used to register the flow of the liquid as a function of time, and freeze-frames at 30 s were used to detect the traveled distance. Three replicates for each sample were carried out.

#### 2.2.2. Microbiological Analyses

After 8 months of room temperature storage, the microbiological counts of Total Aerobic Mesophilic Bacteria (AMB) and the counts of aerobic and anaerobic, either mesophilic or thermophilic spores, were analyzed to ensure product stability and that the products were safe for sensory tests. AMB were determined by Plate Count Agar (PCA) (Merck, Darmstadt, Germany), incubated at 30 ± 1 °C for 72 h, according to the International Organization for Standardization (ISO) method 4833-1:2013 [32].

Spores were quantified by subjecting the sample to a thermal treatment of 80 °C for 10 min for the mesophilic spore counts and to a heat treatment of 100 °C for 5 min for the thermophilic spores [33]. After that, proper sample dilutions were performed in peptone water, and counts were performed in PCA at 30 ± 1 °C for 72 h for Aerobic Mesophilic Spores (A-MS) and at 50 ± 1 °C, 72 h for Aerobic Thermophilic Spores (A-TS). For enumeration of anaerobic spores, Schaedler Anaerobe Agar (Oxoid, Unipath, Basingstoke, UK) media was used, anaerobic incubation at 30 ± 1 °C for 72 h was used for Anaerobic Mesophilic Spores counts (An-MS) and at 50 ± 1 °C, 72 h for Anaerobic Thermophilic Spores counts (An-TS).

All determinations were performed in triplicate. Counts were expressed in log10 (cfu/g). The detection limit for all the count methods was 1 log10 cfu/g.

#### 2.2.3. Sensory Analysis

Sensory quality was evaluated by a trained panel of eight tasters. They all had more than 2 years of experience in the descriptive analysis of different foods. Initially, two open discussion sessions were held to select the sensory attributes to be evaluated by consensus. A total of six odor attributes (global intensity, green leafy vegetables, leek, fishy, legumes/mealy, and shrimp/shellfish), seven taste attributes (global intensity, salty, sweet, umami, fishy, shrimp/shellfish, and bitter) and five textural attributes (creaminess, particles presence, color homogeneity, graininess, and fluidity) were tested. Two additional preliminary sessions were carried out to unify the use of the evaluation scale (0 = absence to 10 = maximum expected intensity in the product under examination) among tasters.

Before serving, the creams were heated in a water bath for approximately 15 min till they reached a product core temperature of 60 °C. Samples were presented in different orders for each taster and session following a Williams Latin square design (balanced for first order and carry-over effects) [34]. Different formulations (including the control sample) were evaluated in each taste session. A total of six sessions were held, considering each of the three production batches, during two consecutive weeks. In each session, the control sample was evaluated together with other three formulations. Each formulation was evaluated at least three times, considering every time a different batch. This six-session evaluation was repeated for the shelf life studies after 8 months of storage. All samples, coded with three-digit random numbers, were analyzed in a standardized tasting room with green light to mask color differences [35]. The tasters were provided with mineral water and golden apple slices to clean their palates between sample tastings. The performance of the panel was verified using the standard methodology [36].

### 2.3. Analysis of the Results

Physicochemical and microbiological data were analyzed by one-way analysis of variance (ANOVA) considering two factors in an independent way: formulation and storage time. The combined effects of these two factors were checked, but no significant interaction was detected.

Significant differences between mean values were assessed using Tukey’s post hoc test. For all statistical analyses, a 95% level of confidence (*p* < 0.05) was used as the threshold for significance. All experimental data were statistically analyzed using XLSTAT^®^ software version 2021.1.1.1110 (Addinsoft, Paris, France). In all these cases, the batch effect was not considered given that a previous assessment showed that batches did not differ significantly among them.

In the case of the sensory data, a two-way ANOVA was performed, thus including the effect of the tasting session as a random factor to generalize the results to any session since. No interaction between the tasting session and the two factors explored (formulation and storage time) was observed (*p* > 0.05).

## 3. Results and Discussion

### 3.1. Nutritional Composition

The nutritional profile of the different cream formulations was computed based on the data available on the labels or technical sheets of the commercial ingredients (Table A1, Appendix A). Energy value was calculated according to the energy factors provided in the EU Regulation n° 1169/2011 [37]. The nutritional values associated with each formulation, calculated from the ingredient data sheet, are reported in Table 2. Results showed that all the formulations with microalgae can be labeled as high in protein according to EU legislation, while the control formulation can only be labeled as a source of protein [27]. Similarly to what was found in other studies, the inclusion of microalgae can be used to improve protein content and generally the nutritional profile of the product [13,21,38,39,40]. The claim “high protein”, particularly, is very important to create an added value product with enhanced appeal for the consumers [41].

### 3.2. Evaluation of Changes Related to Formulation

Characterization of the different vegetable cream formulations is reported in Table 3 (physicochemical properties), Table 4 (olfactory profile), Table 5 (flavor/taste profile), and Table 6 (visual and textural attributes). As a general statement, significant differences were observed between the control and all other formulations across all parameters, except for sensory-evaluated creaminess. The details of these changes are analyzed in Section 3.2.1. No changes associated with formulation were found in the microbiological parameters as shown in Table A3 (Appendix A).

#### 3.2.1. Physicochemical Properties

The results of the physicochemical characterization (Table 3) indicate that the addition of microalgae reduced the consistency of the cream in comparison with the value measured for the control samples. SP formulation was the most similar to the control regarding the consistency measured with the Bostwick consistometer. This is probably due to the low percentage (1%) of Spirulina inclusion. All the other formulations showed thinner consistency compared to the Control, confirming the results from Boukid et al. [21]. One reason could be the decrease in pectins in these formulations as a consequence of the partial substitution of vegetables with microalgae. Since pectins are one of the main components driving the consistency of the cream [42], a change in their concentration could affect this attribute. Other authors observed that microalgae SCI induced a reduction in particle size [43] and resistance to flow [44], contributing to lowering the consistency of the final product [45]. Microalgae inclusion also decreased the pH of the creams, in agreement with Lafarga et al. [22] for broccoli soups and Boukid et al. [21] for vegetable creams, with the exception of *T. chui*, which significantly increased the pH compared to the control samples.

Regarding moisture content, the addition of microalgae ingredients in powder form resulted in a significant decrease in MC% compared to the control samples, due to their ability to retain water. This was observed for all the formulations, except for the SP, possibly as a consequence of the low percentage (1%) of microalgae (Spirulina) inclusion. Results are in accordance with previously mentioned studies [21,22].

**Figure 1:**
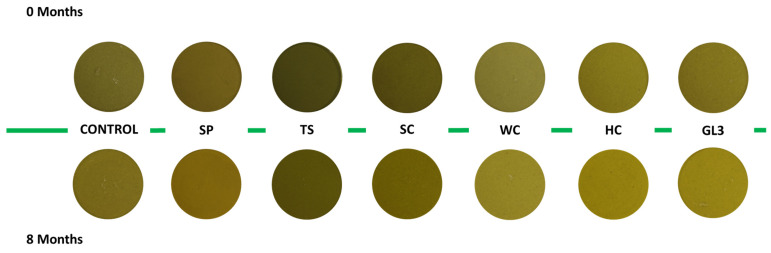
Picture of the different cream formulations at time 0 and after 8 months of storage.

Microalgae ingredients affected the color of the creams, with significant effects on the CIELab parameters in the range of detection of the human eye (ΔE > 3), as clearly observable in Figure 1. Furthermore, looking at the picture of the different ingredients (Figure A1, Appendix A), it is possible to observe how the difference in color of the raw material is reflected by the obtained results. The yellow species increased significantly their yellow hues, resulting in a higher overall impact on color. The inclusion of “Honey” *C. vulgaris* generated the greatest total color variation (ΔE = 10.87) compared to the control samples, while the addition of Spirulina had, globally, the lowest effect on this parameter (ΔE = 3.36). Interestingly, SP was also the only formulation showing an increase in a* (higher redness and lower green coloration), while yellowness (b*) increased in all the formulations added with microalgae, except in the case of TS (*T. chui*). Lightness (L*) decreased in formulations prepared with green microalgae ingredients (SP, TS, SC). This is in accordance with the results observed by Lafarga et al. [22] in broccoli soups, which also correlated a reduction in L* with a decrease in vegetable cream acceptability. On the contrary, in formulations prepared with yellow microalgae ingredients (HC, WC, and GL3), as observable in Table 3, L* significantly increased, indicating a potential positive impact on the visual appeal of the final product [46]. The contemporary changes in the yellow hues and lightness in these formulations could be evaluated positively or not by the consumers, depending on their habits. However, variations from green color in vegetable products are usually associated with ripeness and could be carefully investigated when developing a product that wants to reach the market [47].

The addition of the microalgae ingredients caused an overall positive effect on the stability of the cream emulsion, by significantly reducing the Sy%, with the only exception of samples formulated with *T. chui*, which did not differ from the Control. Similar results were found by Barkallah et al. [48] in yogurts fortified with Spirulina, which showed lower percentages of syneresis in samples containing higher concentrations of microalgae. These results could be justified by the higher amounts of protein in samples fortified with microalgae compared to the control (see Table 2), since proteins may establish, especially at acidic pHs, intermolecular interactions and physical entanglements resulting in a more stable structure of the cream matrix [44]. Furthermore, Du et al. [49] demonstrated that, after high-temperature treatments, protein can also form covalent complexes with pectin, which may further reduce protein precipitation phenomena and increase the emulsion stability.

All the formulations added with microalgae ingredients had higher concentrations of soluble solids (°Brix) than the control samples. This effect could be a consequence of the thermal treatments, which promoted the solubilization of water-soluble compounds because of the induced algae-cell breaking and intracellular material leakage [50].

#### 3.2.2. Sensory Properties

Significant differences between the control and the other formulations were detected in the attributes linked to the olfactory profile (Table 4). The panelists perceived significantly lower notes of “green leafy vegetables” and “leek” in all the formulations with microalgae ingredients. In the case of the formulation containing *T. chui*, the “legumes/mealy” attribute was perceived as significantly lower than in the control product. “Fishy” and “shrimp/shellfish” smells were also associated with formulations containing microalgae ingredients, with the highest scores observed in the samples added with *T. chui*. It could be hypothesized that these fishlike attributes could partially mask the vegetable and legume-like notes in all the formulations containing microalgae.

Global taste intensity and “umami” attribute were perceived significantly more intensely in samples added with microalgae ingredients than in control formulation (Table 5). As a general trend, the inclusion of microalgae also increased the perception of “salty” and “bitter” attributes, even if not always significantly, if compared to the control samples. Panelists noticed a decrease in the “sweet” attribute in formulations containing “smooth” or “white” *C. vulgaris*. Samples containing microalgae ingredients obtained the highest scores for “fishy” and “shrimp/shellfish” tastes, which were extremely intense in the formulation added with *T. chui*. Noticeably, the TS formulation had the highest scores for “global taste intensity”, “salty”, “umami”, “fishy”, “shrimp/shellfish”, and “bitter” attributes, underlying the challenging organoleptic profile of this species.

According to the previous literature, microalgae sensory profile is characterized by fishy notes, which can lead to sensory-related food aversion and incentive food neophobia, negatively affecting consumer evaluation [51,52,53]. Considering this, the formulations with the lowest intensity in fishy and shrimp/shellfish flavors, GL3 and WC, followed by SP and HC, have more potential for market launch. Moreover, as they presented increased salty and umami perception, a possible use of Spirulina and *C. vulgaris* (White, Honey, and GL3) as natural flavor enhancers is suggested.

These differences in the sensory profile of the cream (Table 4 and Table 5) can be associated with the biomass composition of the different microalgae species/strains. Microalgae’s unique aromas and flavors arise from a variety of volatile organic compounds (VOCs) produced as secondary metabolites. The fishlike odor detected in this study is a result of VOCs generated during the polyunsaturated fatty acids (PUFAs) degradation, such as hydrocarbons, alcohols, aldehydes, esters, and carboxylic acid [54]. In addition, Van Durme et al. [55] reported that saltwater species like *T. chui* are characterized by the presence of sulfuric compounds that impart the characteristic shellfish and marine flavors. However, depending on their molecular weight and saturation level, these compounds can emit a range of aromas, not always unpleasant. *C. vulgaris*, for example, has been reported to have a high content of aldehydes, conferring a grassy, vegetable, and/or fruity taste [56], which are in line with the milder sensory characteristics observed for these species in the present study.

Furthermore, amino acids are also an important class of taste-active compounds in aquatic foods. Relatively high contents of glutamic acid have been associated with the perception of umami and salty tastes in foods [57] in Spirulina, *T. chui*, and *C. vulgaris* single-cell ingredients [58], indicating that their inclusion in the food matrix may have a significant impact on the taste of the final products. Tryptophan could be responsible for the sweet taste of spirulina, while the bitter taste perceived by the panel in this study might be related to Valine, Histidine, and Arginine content in microalgae [59].

The difference in the content in all these compounds influenced the sensory profile of the formulations.

Moving to the results about visual appearance and textural perception (Table 6), no significant difference in creaminess was observed between the different formulations. SP was the only formulation where the presence of particles was perceived with a significantly lower intensity score compared to all the other formulations (including the Control). This was also reflected in the score of the perceived graininess, which was the lowest in the case of SP, with a significant difference from the intensity perceived for the Control. Moreover, two formulations containing microalgae ingredients (i.e., SP and TS) showed a significantly higher color homogeneity compared to the other samples. This could be attributed to the dark-green color of the single-cell microalgae ingredients (from *C. vulgaris* and *T. chui*, respectively), which adjusted better with the color of the other vegetal ingredients. The inclusion of *T. chui* also decreased graininess and increased fluidity compared to the control formulation. A general trend toward increased values of fluidity (Table 6) was found for all the samples added with the microalgae ingredients, in agreement with the results from Bostwick test that showed a reduced consistency of these samples compared to the control ones. Results about sensory perceived texture are in line with other studies, which revealed the ability of microalgae to stabilize emulsions and improve product homogeneity [44,60,61].

The changes in the textural attributes of the microalgae-added formulations perceived by the panel (Table 6) could be interesting or not depending on the desired properties of the final product. The SP formulation, for example, could be interesting for its homogeneity, reduced graininess, and particle perception, but it might also be perceived as more processed and far from the traditional handcrafted version by consumers.

### 3.3. Quality over Time Investigation

Physicochemical properties (color, moisture content, consistency, pH, °Brix, syneresis), microbiological parameters, and sensory profile changes were investigated considering 8 months of storage time, as described in Section 2.2. According to the microbiological tests, the product proved to be safe for consumption after 8 months. The results are reported in Table A3 (Appendix A). Microbial loads were always under 2 Log (ufc/g) and in most cases even under the limit of detection of 1 Log (ufc/g). Moreover, only slight changes in pH were observed during the storage, ensuring that no consistent microbial growth took place (see Table 7). The microbiological stability aspect was not previously taken into account in the other studies about creams reformulation with microalgae.

On the other hand, the results for the physicochemical and sensory properties of the creams as a function of time are shown in Table 7 and Table 8, respectively, taking into account the data at time 0 and 8 months. As already explained in Section 2.3, no influence of the microalgae inclusion or species was detected; for this reason, the results are presented only as a function of time.

All the physicochemical parameters showed significant differences except water content (WC%) and °Brix. A slight reduction of the pH, lightness (L*), and syneresis values as a function of time can be observed, while consistency, redness (a*), and yellowness (b*) increased. This can also be observed in Figure 1, where the pictures of the different formulations are shown. Particularly important are the color modifications.

Color and appearance are crucial for attracting consumers to vegetable products, as they are associated with freshness and flavor quality [47]. Maintaining these attributes is essential for product appeal. Greenness is a key quality measure in heated vegetables, reflecting consumer preferences [62]. In the present study, storage time significantly influences color with a simultaneous reduction in L* and increase in a* and b* parameters. Moreover, these changes can be associated with an increase in the brown color as defined by Pathare et al. [63]. Phenomena like yellowish (b* increase) and browning, which occurred in the cream (also visible in Figure 1), should be controlled because they are associated with ripeness and can reduce visual appeal [64].

Color changes in the product were likely due to the instability of its main pigments, which are sensitive to acid pH and the presence of oxygen, as supported by the literature [65,66]. Additionally, chemical transformations of bioactive compounds, protein degradation, and lipid oxidation could have affected both color and pH [58,67,68]. Enzyme activity can be excluded or considered limited due to inactivation during autoclaving, as well as microbial activity, which was limited according to the observed reduced growth.

On the other hand, the increase in consistency during storage could be related to particle aggregations, flocculation, and emulsion stabilization over time [69,70].

In Table 8, the ANOVA for the odor, flavor/taste, and visual/textural attributes are reported.

Regarding odor profile, a significant decrease in fishy and shrimp/shellfish odor and a concurrent increase in green leafy and mealy odor intensity with time were observed. Despite this, no changes in the global intensity occurred. Talking about the flavor/taste attributes, a decrease in the global intensity of taste was detected. This was mainly related to an important decrease in fishy flavor. In this case, the reduction in fishy smell and taste can contribute to enhancing the consumers acceptance, enhancing the vegetable and legumes notes as observable in Table 8. Other changes like the variations in texture (creaminess, particle presence, etc) should be investigated deeper because, depending on the consumer, they can be perceived positively or negatively as previously discussed in Section 3.2.2. The other flavor/taste attributes did not show significant differences over storage time.

Visual/textural evaluation showed an increase in creaminess (in agreement with the results obtained with Bostwick measurements) in contrast with a contemporary increase in fluidity over time. Moreover, the sensation of particle presence and graininess decreased. No changes in color homogeneity were observed, which is beneficial as this attribute was demonstrated to influence consumer satisfaction [71].

As reported by other studies, the loss in sensory properties over time could occur as a consequence of degradation of ester and aldehyde compounds and/or the formation of hydrophobic amino acid residues as a consequence of residual enzyme activity, oxidation, or thermal degradation during sterilization [72,73].

## 4. Conclusions

This study highlights the promising potential use of microalgae species, in the formulation of high-protein vegetable creams. While minor differences in physicochemical parameters were observed due to microalgae inclusion, the enhanced emulsion stabilization and homogenization effects suggest valuable technological applications for the food industry. Importantly, the inclusion of microalgae did not affect the shelf life or storage behavior, indicating their suitability for long-term storage food reformulation. However, the variation in color over time needs to be managed to meet consumer preferences and keep browning phenomena under control. Regarding sensory aspects, the findings emphasize the critical role of species selection in food formulation, as their properties can substantially differ. Due to their composition, microalgae ingredients significantly improved umami and salty flavors, positioning them as natural salt enhancers. However, to maintain the cream’s natural flavor, it is essential to manage off-colors, odors, and flavors. Yellow chlorellas emerged as the least impactful in terms of flavor, though their influence on color deserves further investigation to assess consumer perception. Conversely, the marine species *Tetraselmis chui* notably altered the sensory profile, imparting a strong salty and fishy taste, which could be advantageous for developing new food concepts better than reformulating familiar ones.

Looking ahead, future research should explore additional nutritional benefits of these creams, such as antioxidant capacity, digestibility, and vitamin content. Investigating the release of polysaccharides, proteins, and lipids during thermal processes could also provide useful insights. Moreover, advancing sensory evaluation through the chemical identification of compounds linked to sensory changes will be crucial. This approach can help develop effective mitigation strategies, essential for the widespread acceptance of microalgae-based foods by both industry and consumers. In this regard, providing information that can explain the price premium and reduce neophobia about microalgae products was revealed to be crucial to improve consumer acceptance [74]. On the other hand, as reported by Lamanna et al. [75], to increase consumer engagement, it is also important to leverage social media to combat misinformation and enhance public understanding.

## Figures and Tables

**Table 1:** Samples composition (g/100 g fresh weight basis).

Ingredient	Control	SP	TS	SC	WC	HC	GL3
Spirulina	0.0	1.0	0.0	0.0	0.0	0.0	0.0
*Tetraselmis chui*		0.0	2.0				
Smooth *Chlorella*			0.0	4.0			
White *Chlorella*				0.0	2.0		
Honey *Chlorella*					0.0	4.0	
*Chlorella GL3*						0.0	4.0
Spinach	15.4	15.2	14.5	14.5	14.5	14.5	14.5
Zucchini	13.5	13.3	13.1	12.7	13.1	12.7	12.7
Chickpea							
Leek	9.7	9.5	9.4	9.10	9.40	9.10	9.10
Broccoli							
Chard	5.8	5.7	5.6	5.4	5.6	5.4	5.4
Water	29.0						
Oil	2.9						
Salt	0.5						

**Table 2:** Nutritional values of the different formulations.

Sample	Energy (kcal/100 g)	Carbohydrates (g/100 g)	Proteins (g/100 g)	Fats (g/100 g)	Fiber (g/100 g)	%Kcal Protein
Control	57.02	3.75	2.50	3.08	2.19	17.51%
SP	60.44	3.91	3.04	3.17	2.19	20.12%
TS	62.83	3.95	3.34	3.23	2.35	21.26%
SC	67.18	4.98	4.98	3.30	2.87	20.01%
WC	62.02	4.81	2.95	3.19	2.53	20.13%
HC	68.21	5.80	3.56	3.30	2.06	20.59%
GL3	71.56	5.07	3.70	3.48	2.59	20.71%

**Table 3:** Physicochemical properties of vegetable creams formulations. Different letters in the same column indicate significant (*p* < 0.05) differences between samples for the given parameter.

	Bostwick (cm)	pH	L*	a*	b*	ΔE	MC%	Sy%	°Brix
Control	5.87 ^d^	5.75 ^b^	43.78 ^b^	−1.64 ^b^	21.66 ^e^	0.54 ^f^	87.52 ^a^	68.02 ^a^	4.05 ^c^
SP	6.39 ^cd^	5.73 ^c^	41.91 ^c^	0.71 ^a^	22.99 ^d^	3.36 ^e^	86.70 ^ab^	57.50 ^cd^	5.00 ^b^
TS	6.98 ^bc^	5.99 ^a^	37.40 ^e^	−1.94 ^cd^	15.78 ^f^	8.69 ^b^	85.87 ^bc^	63.30 ^ab^	5.30 ^ab^
SC	8.12 ^a^	5.70 ^e^	39.14 ^d^	−1.83 ^bc^	21.72 ^e^	4.73 ^d^	84.21 ^d^	57.00 ^d^	5.70 ^a^
WC	7.08 ^bc^	5.72 ^de^	47.46 ^a^	−2.15 ^d^	24.54 ^c^	4.74 ^d^	85.76 ^bc^	60.11 ^bcd^	5.02 ^b^
HC	7.58 ^ab^	5.72 ^d^	47.62 ^a^	−2.06 ^cd^	31.81 ^a^	10.9 ^a^	84.15 ^d^	56.83 ^d^	5.20 ^b^
GL3	6.97 ^bc^	5.68 ^f^	46.63 ^a^	−1.91 ^c^	27.86 ^b^	6.86 ^c^	85.57 ^c^	62.60 ^bc^	4.87 ^b^

**Table 4:** Olfactory attributes scores of vegetable cream formulations. Different letters in the same column indicate significant (*p* < 0.05) differences between samples for the given parameter.

	Global Odor Intensity	Green Leafy Vegetables	Leek	Fishy	Legumes/Mealy	Shrimp/Shellfish
Control	6.34 ^c^	5.71 ^a^	5.82 ^a^	0.00 ^d^	3.27 ^a^	0.00 ^d^
SP	6.57 ^bc^	4.53 ^b^	3.74 ^b^	0.72 ^bcd^	2.99 ^a^	0.79 ^bc^
TS	8.38 ^a^	2.66 ^c^	1.59 ^c^	5.95 ^a^	1.13 ^b^	4.86 ^a^
SC	6.85 ^b^	4.23 ^b^	3.19 ^b^	1.75 ^b^	2.73 ^a^	1.33 ^b^
WC	6.47 ^bc^	4.70 ^b^	3.97 ^b^	0.66 ^cd^	2.78 ^a^	0.67 ^bc^
HC	6.54 ^bc^	4.73 ^b^	3.63 ^b^	1.17 ^bc^	2.67 ^a^	0.95 ^bc^
GL3	6.17 ^c^	4.54 ^b^	3.78 ^b^	0.25 ^cd^	2.63 ^a^	0.16 ^cd^

**Table 5:** Flavor/taste attributes scores of vegetable cream formulations. Different letters in the same column indicate significant (*p* < 0.05) differences between samples for the given parameter.

	Global Taste Intensity	Salty	Sweet	Umami	Fishy	Shrimp/Shellfish	Bitter
Control	5.94 ^d^	4.11 ^d^	2.68 ^a^	1.42 ^c^	0.00 ^d^	0.00 ^d^	2.31 ^d^
SP	6.59 ^c^	4.84 ^bc^	2.14 ^ab^	3.31 ^b^	1.28 ^bc^	1.67 ^b^	2.99 ^bcd^
TS	8.72 ^a^	7.14 ^a^	0.74 ^c^	4.66 ^a^	6.74 ^a^	5.44 ^a^	4.44 ^a^
SC	7.17 ^b^	5.31 ^b^	1.85 ^b^	3.56 ^b^	2.08 ^b^	1.92 ^b^	4.01 ^ab^
WC	6.46 ^c^	4.54 ^cd^	2.25 ^ab^	2.70 ^b^	0.89 ^c^	0.96 ^bc^	2.75 ^cd^
HC	6.81 ^bc^	5.07 ^bc^	2.09 ^ab^	3.33 ^b^	1.44 ^bc^	1.54 ^bc^	3.44 ^bc^
GL3	6.45 ^c^	5.02 ^bc^	2.56 ^ab^	2.63 ^b^	0.49 ^cd^	0.57 ^cd^	2.69 ^cd^

**Table 6:** Visual and textural attributes scores of vegetable cream formulations. Different letters in the same column indicate significant (*p* < 0.05) differences between formulations for the given parameter.

	Creaminess	Particle Presence	Color Homogeneity	Graininess	Fluidity
Control	5.93	3.94 ^a^	6.92 ^b^	5.26 ^abc^	5.25 ^c^
SP	5.92	2.78 ^b^	8.53 ^a^	2.91 ^e^	5.84 ^abc^
TS	5.66	3.72 ^a^	8.01 ^a^	3.78 ^d^	6.30 ^a^
SC	6.18	4.23 ^a^	7.11 ^b^	5.34 ^ab^	5.33 ^c^
WC	5.81	3.75 ^a^	7.04 ^b^	4.64 ^bc^	5.66 ^abc^
HC	5.97	4.13 ^a^	6.60 ^b^	4.57 ^c^	6.10 ^ab^
GL3	6.02	3.85 ^a^	6.58 ^b^	5.46 ^a^	5.37 ^bc^

**Table 7:** Physicochemical properties of the creams at time 0 and 8 months. Different letters in the same column indicate significant (*p* < 0.05) differences between formulations for the given parameter.

	Bostwick (cm)	pH	L*	a*	b*	Δ	WC%	Sy%	°Brix
0 months	7.17 ^a^	5.91 ^a^	43.7 ^a^	−1.67 ^a^	23.4 ^b^	0.00 ^b^	85.6	64.1 ^a^	5.08
8 months	6.82 ^b^	5.75 ^b^	43.2 ^b^	−1.42 ^b^	24.2 ^a^	6.06 ^a^	85.7	57.4 ^b^	4.96

**Table 8:** Sensory attributes scores of creams at times 0 and 8 months. Different letters in the same column indicate significant (*p* < 0.05) differences between formulations for the given parameter.

Olfactory Attributes							
	Global Intensity		Green Leafy Vegetables	Leek	Fishy	Legumes/Mealy	Shrimp/Shellfish
0 months	6.69		4.21 ^b^	3.76	1.70 ^a^	2.34 ^b^	1.28 ^a^
8 months	6.83		4.67 ^a^	3.58	1.28 ^b^	2.85 ^a^	1.18 ^b^
**Flavor/Taste Attributes**							
	Global intensity	Salty	Sweet	Umami	Fishy	Shrimp/ shellfish	Bitter
0 months	6.99 ^a^	5.15	2.13	3.11	2.25 ^a^	1.82	3.17
8 months	6.77 ^b^	5.14	1.96	3.06	1.38 ^b^	1.58	3.30
**Visual/Textural Attributes**							
	Creaminess		Particle Presence	Color Homogeneity		Graininess	Fluidity
0 months	5.57 ^b^		4.10 ^a^	7.15		4.86 ^a^	5.36 ^b^
8 months	6.29 ^a^		3.44 ^b^	7.37		4.27 ^b^	6.03 ^a^

## Data Availability

The original contributions presented in the study are included in the article, further inquiries can be directed to the corresponding author.

## Appendix A

**Table A1:** Chemical composition of ingredients used in vegetable cream formulations.

Ingredients	Energy, (kcal/100 g)	Fat (g/100 g)	Saturated Fatty Acids (g/100 g)	Carbohydrates (g/100 g)	Sugars (g/100 g)	Dietary Fibers (g/100 g)	Protein (g/100 g)	Salt (NaCl) (g/100 g)
Spinach	30.0	0.50	0.10	1.10	0.90	3.40	3.60	0.20
Zucchini	25.0	0.30	0.10	2.60	2.50	1.30	2.40	0.00
Chickpea	13.1	2.00	0.30	17.7	1.00	6.20	7.40	0.00
Leek	33.0	0.30	0.00	4.20	3.90	3.10	1.80	0.00
Broccoli	30.0	0.40	0.10	2.30	2.20	2.90	2.90	0.00
Chard	30.0	0.30	0.00	3.60	0.70	1.30	2.80	0.50
Water	0.00	0.00	0.00	0.00	0.00	0.00	0.00	0.00
Sunflower oil	81.0	90.0	9.80	0.00	0.00	0.00	0.00	0.00
Salt	0.00	0.00	0.00	0.00	0.00	0.00	0.00	98.0
Spirulina	38.8	7.70	2.70	20.3	3.10	3.20	57.5	0.50
*Tetraselmis chui*	33.6	7.90	7.63	15.5	4.30	11.2	45.3	1.40
Smooth *Chlorella*	30.0	6.00	1.80	36.0	0.00	20.0	36.0	0.35
White *Chlorella*	29.6	5.92	1.48	57.8	4.00	20.0	26.3	0.30
Honey *Chlorella*	35.0	6.00	1.80	56.0	2.50	9.00	30.0	0.25
GL3 strain *Chlorella*	40.8	10.5	0.00	38.2	1.00	13.1	33.5	0.20

**Table A2:** Autoclave sterilization program.

Phase	Time (min)	Temperature (°C)	Pressure (mbar)
1	15	116	2213
2	74	116	2213
3	10	30	2213
4	10	30	1013

**Table A3:** Values of the samples microbiological load (Log CFU/g) after 8 months of storage for Aerobic Mesophilic Bacteria (A-MB), Aerobic Mesophile Spores (A-MS), Aerobic Thermophile Spores (A-TS), Anaerobic Mesophile Spores (An-MS), and Anaerobic Thermophile Spores (An-TS).

Sample	Batch	Replicate	pH	A-MB	A-MS	A-TS	An-MS	An-TS
Control	1	1	5.68	0.0	0.0	0.0	1.0	0.0
	1	2	5.66	0.0	0.0	0.0	0.0	0.0
	2	1	5.71	0.0	0.0	0.0	0.0	0.0
	2	2	5.67	0.0	0.0	0.0	0.0	0.0
	3	1	5.67	0.0	0.0	0.0	0.0	0.0
	3	2	5.66	1.0	0.0	0.0	0.0	0.0
SP	1	1	6.16	0.0	0.0	0.0	0.0	0.0
	1	2	6.16	0.0	0.0	0.0	0.0	0.0
	2	1	6.17	0.0	0.0	0.0	0.0	0.0
	2	2	6.17	0.0	0.0	0.0	0.0	0.0
	3	1	6.16	0.0	0.0	0.0	0.0	0.0
	3	2	6.14	0.0	0.0	0.0	0.0	0.0
TS	1	1	5.93	0.0	0.0	0.0	2.0	0.0
	1	2	5.92	0.0	0.0	0.0	0.0	0.0
	2	1	5.93	0.0	0.0	0.0	0.0	0.0
	2	2	5.92	0.0	0.0	0.0	0.0	0.0
	3	1	5.92	0.0	0.0	0.0	0.0	0.0
	3	2	5.93	1.3	0.0	0.0	0.0	0.0
SC	1	1	5.63	0.0	0.0	0.0	0.0	0.0
	1	2	5.62	0.0	0.0	0.0	1.3	0.0
	2	1	5.62	0.0	0.0	0.0	0.0	0.0
	2	2	5.62	0.0	0.0	0.0	0.0	0.0
	3	1	5.63	0.0	0.0	0.0	0.0	0.0
	3	2	5.63	1.0	0.0	0.0	0.0	0.0
WC	1	1	5.64	0.0	0.0	0.0	0.0	0.0
	1	2	5.65	0.0	0.0	0.0	0.0	0.0
	2	1	5.66	0.0	0.0	0.0	0.0	0.0
	2	2	5.65	0.0	0.0	0.0	0.0	0.0
	3	1	5.68	0.0	0.0	0.0	0.0	0.0
	3	2	5.64	1.0	0.0	0.0	0.0	0.0
HC	1	1	5.64	0.0	1.0	0.0	0.0	0.0
	1	2	5.64	0.0	0.0	0.0	0.0	0.0
	2	1	5.63	0.0	0.0	0.0	0.0	0.0
	2	2	5.67	0.0	0.0	0.0	0.0	0.0
	3	1	5.66	0.0	0.0	0.0	1.0	0.0
	3	2	5.65	0.0	0.0	0.0	0.0	0.0
GL3	1	1	5.58	1.3	0.0	1.0	0.0	0.0
	1	2	5.59	0.0	0.0	0.0	0.0	0.0
	2	1	5.60	0.0	0.0	0.0	0.0	0.0
	2	2	5.59	0.0	0.0	0.0	0.0	0.0
	3	1	5.60	1.0	0.0	0.0	0.0	0.0
	3	2	5.59	0.0	0.0	0.0	0.0	0.0
